# Authors’ response to Hartwig and Davies

**DOI:** 10.1093/ije/dyw241

**Published:** 2016-09-20

**Authors:** From John P Kemp, Adrian Sayers, George Davey Smith, Jonathan H Tobias, David M Evans

**Affiliations:** ^1^University of Queensland Diamantina Institute, Translational Research Institute, Brisbane, QLD, Australia; ^2^MRC Integrative Epidemiology Unit and; ^3^School of Clinical Sciences, University of Bristol, Bristol, UK

In their letter, Hartwig and Davies^1^ raise an important issue that was not discussed in the original Mendelian randomization Egger regression (MR Egger) paper by Bowden *et al.*^2^ Hartwig and Davies point out that, similar to other varieties of MR, MR-Egger is also susceptible to weak instrument bias. In the case of single-sample MR-Egger, this means that estimates of the causal effect may be biased towards the observational association when weak instruments are used in the analysis (as is the case with traditional single-sample MR). Hartwig and Davies also point out a potential solution to this problem, the utilization of more precise externally derived estimates of the relevant single nucleotide polymorphism (SNP)-exposure association (i.e. from larger publicly available genome-wide association meta-analyses).

We agree with Hartwig and Davies’ conclusions that weak instrument bias is a problem in MR-Egger as it is in traditional MR analyses, and that it is critically important that users of the technique are aware of this possibility. Hartwig and Davies also refer to a recent study of ours^3^ in the *International Journal of Epidemiology*, where we used several different types of MR analyses (including MR-Egger) to examine a possible causal effect of adiposity on bone mineral density (BMD). As Hartwig and Davies fairly acknowledge in their letter, not only did we discuss the possibility of weak instrument bias in our study, we also performed preliminary simulations to investigate its effect on MR-Egger (the results of which broadly agree with their assertions). As Hartwig and Davies note, MR-Egger was only one small component of our paper and none of our key results (which we believe to be robust) rely on the results of these analyses in isolation, and indeed many of the other analyses reported in our paper do not suffer from potential bias due to weak instruments.

Hartwig and Davies did, however, suggest that we could have used estimates from an external source to obtain less biased results in our MR-Egger analyses. Whereas we agree that this would be good practice in most situations, we do not feel that it would have been appropriate in our study, for two reasons. First, the focus of our article was not on a possible causal relationship between body mass index and BMD (which is well-known and widely accepted), but rather on a possible causal relationship between adiposity [as operationalized as fat mass calculated from total body dual-energy X-ray absorptiometry (DXA)] and BMD. There are no publicly available genome-wide association studies of total body fat mass as measured by total body DXA, and therefore no external estimates that we could have applied in our analyses (i.e. as far as we are aware, we are currently the largest such study). We could have used external estimates for analyses involving body mass index, but this would have been of limited utility since body mass index is a far from perfect measure of adiposity. Second, our study involved 9-year-old children from the Avon Longitudinal Study of Parents and Children. It is unclear the extent to which effect sizes of adiposity-associated variants in adults reflect effect sizes of adiposity-associated variants in children (as Hartwig and Davies recognize), and we therefore feel it would have been inappropriate to use adult-derived external estimates in our study of children.
